# Imposters, Bots, and Other Threats to Data Integrity in Online Research: Scoping Review of the Literature and Recommendations for Best Practices

**DOI:** 10.2196/70926

**Published:** 2025-08-29

**Authors:** Isabella B Strickland, Amy K Ferketich, Alayna P Tackett, Joanne G Patterson, Nicholas J K Breitborde, Jade Davis, Megan Roberts

**Affiliations:** 1 College of Public Health, The Ohio State University, 1841 Neil Ave, Columbus, OH, 43210, United States, 1 6142924647; 2 Center for Tobacco Research, The Ohio State University Comprehensive Cancer Center, The Ohio State University, Columbus, OH, United States; 3 Division of Medical Oncology, Department of Internal Medicine, College of Medicine, The Ohio State University, Columbus, OH, United States; 4 Psychiatry and Behavioral Health, College of Medicine, The Ohio State University, Columbus, OH, United States

**Keywords:** review, fraud, data integrity, bots, online data collection, PRISMA

## Abstract

**Background:**

Threats to data integrity have always existed in online human subjects research, but it appears these threats have become more common and more advanced in recent years. Researchers have proposed various techniques to address satisficers, repeat participants, bots, and fraudulent participants; yet, no synthesis of this literature has been conducted.

**Objective:**

This study undertakes a scoping review of recent methods and ethical considerations for addressing threats to data integrity in online research.

**Methods:**

A PubMed search was used to identify 90 articles published from 2020 to 2024 that were written in English, that discussed online human subjects research, and that had at least one paragraph dedicated to discussing threats to online data integrity.

**Results:**

We cataloged 16 types of techniques for addressing threats to online data integrity. Techniques to authenticate personal information (eg, videoconferencing and mailing incentives to a physical address) appear to be very effective at deterring or identifying fraudulent participants. Yet such techniques also come with ethical considerations, including participant burden and increased threats to privacy. Other techniques, such as Completely Automated Public Turing test to tell Computers and Humans Apart (reCAPTCHA; Google LLC), scores, and checking IP addresses, although very common, were also deemed by several researchers as no longer sufficient protections against advanced threats to data integrity.

**Conclusions:**

Overall, this review demonstrates the importance of shifting online research protocols as bots and fraudulent participants become more sophisticated.

## Introduction

Recent years have witnessed a shift in research protocols, with many studies that were previously conducted in-person being moved online [1]. This shift has had several benefits for researchers in terms of easier sampling, broader reach, and better access to historically marginalized populations [2,3]. However, the shift has also ushered in critical concerns about threats to data integrity. While concerns about data integrity have always existed, even with in-person studies, there has been a notable increase in the number and types of threats to data integrity in online studies since the COVID-19 pandemic [4,5].

As outlined in Table 1, the types of threats to data integrity in online research come in several forms. First, satisficers (also known as speeders, straightliners, and cheaters) are individuals who rush through surveys with little care for the accuracy or thoroughness of their responses. While satisficers also exist with study protocols administered in-person or by mail [6,7], this threat is challenging to monitor online. Next, there are repeat participants (also known as duplicate participants). These are individuals who complete screener surveys or study protocols multiple times. Motivations for repeat participation vary: some people may be curious about what happens if they complete the survey with different answers [8]. Others may try to complete the survey multiple times to get extra compensation [9]. Whatever the motivation, this behavior can have serious consequences for data integrity and research findings [10]. And, again, it is often easier for individuals to engage in these behaviors with online research studies.

**Table 1.** Types of threats to data integrity in online research.

Type of threat	Other terms	Definition
Satisficers	Cheaters, straightliners, speeders, and careless participants	Inattentive participants who speed through surveys often not paying attention to questions and responding thoughtlessly
Repeat participants	Duplicate participants	Participants who attempt to complete a study more than once out of curiosity or a desire for additional remuneration
Bots	Chatbots and artificial intelligence respondents	Computer algorithms deployed on studies in order to gain compensation without human effort for completion
Fraudulent participants	Imposters, scammers, bad actors, and lying participants	Participants who lie about their identity or otherwise attempt to deceive researchers often with the intent of gaining study compensation

Another growing threat to data integrity is bots (also known as chatbots and artificial intelligence respondents). Bots are automated computer programs that people can create to randomly and methodically complete online surveys, usually numerous times, to gain compensation without having to take the time and effort to manually complete the survey [11]. Tools such as a Completely Automated Public Turing test to tell Computers and Humans Apart (CAPTCHA; or the newer version, reCAPTCHA; Google LLC) can help prevent bots from infiltrating surveys; however, more advanced bots can bypass these measures [12]. Bots can very quickly complete surveys and, if they are not properly blocked, can compromise results and force time-consuming and expensive relaunches of research projects [13,14].

Finally, there are fraudulent participants (also known as imposter participants, scammers, and lying participants) [4,5,15]. Unlike bots, fraudulent participants are real people who complete a study protocol. However, they lie about themselves to qualify for study participation. For example, Pellicano et al [4] describe a situation where fraudulent participants posed as either people with autism or parents of children with autism during online, qualitative interviews. In this particular example, several clues aroused the researcher’s suspicion, such as keeping cameras off, inconsistent responses between prescreening and the interview, similarities in voices and mannerisms across interviews, and repeated inquiries about payments. Fraudulent participants have been detected in many domains of research but seem, concerningly, to have the largest impact on research on small populations that are often historically minoritized or otherwise vulnerable [10]. One study interviewing research participants found that, on average, 55% of participants who had participated in some sort of research fraud reported fabricating information to qualify for studies [16]. Several studies have found that these participants often respond differently than authentic participants, potentially influencing the results of research studies or weakening the effects detected [17-19].

While many researchers have published concerns or potential solutions to these various threats to data integrity, there has not yet been a review or synthesis of the literature. This research gap makes it difficult and time-consuming for researchers designing new online studies to decide on best practices. Due to the research interests of the authors, we were particularly motivated to identify research methods being used to address threats to online data integrity in the medical and public health domains. Therefore, we used PubMed to conduct a scoping review of methods that address contemporary threats to online data integrity, with keywords that focus on bots and fraudulent participants. Our objectives were to catalog and evaluate the most common research methods used to address these threats and discuss the ethical considerations raised about the techniques. Ultimately, this review aims to expand and centralize knowledge on addressing threats to data integrity in online studies, with the goal of aiding researchers in developing robust online methodologies.

## Methods

### Search Strategy

This search was conducted using Covidence (Veritas Health Innovation), an online tool used to conduct and organize literature reviews. We searched PubMed for the terms “fraud* OR imposter* OR scam* OR bot OR bots.” To be eligible for review, papers needed to be published in or after 2020 because we were interested in methods for addressing the recent threats to data integrity that have emerged since COVID-19. Additional eligibility criteria were: discussing an online study, using human participants, being written in English, and having at least one paragraph dedicated to discussing threats to data integrity.

Our PubMed search yielded an initial 10,681 publications. After eliminating 189 duplicate texts using Covidence, 10,492 publications remained to be screened. Our first phase of screening checked all abstracts and eliminated those not pertaining to online research. Of the 10,492 publications, 196 were retained. In the second phase of screening, the full article was checked for inclusion criteria. Ultimately, 90 articles met criteria for inclusion in this review [2-4,11,13,15,20-103]. This review followed the PRISMA (Preferred Reporting Items for Systematic Reviews and Meta-Analyses) 2020 guidelines.

### Data Extraction

A codebook was developed through an interrogative process. First, a codebook was created a priori according to our research questions. Additional codes and subcodes were added through an inductive process. Two team members (IBS and JD) independently reviewed and coded each of the 90 articles [2-4,11,13,15,20-103]. A senior team member (MR) reviewed their interrater agreement and resolved all discrepancies.

The following information was extracted and coded from the final 90 articles [2-4,11,13,15,20-103]: article type (eg, original research and commentary), type of data collection (eg, qualitative and quantitative), methodology of the study (eg, survey and qualitative interview), country where the study was conducted, recruitment methods (eg, social media and survey service platform), type of suspected threat to data integrity (eg, bots and fraudulent participants), the estimated prevalence of compromised data, and techniques mentioned for addressing threats to data integrity (eg, authenticating personal information and attention checks).

Techniques to address threats to data integrity were additionally sorted into 3 categories. “Very effective” techniques were those that authors of the reviewed articles, especially in the most recent publications, deemed to be successful at identifying poor-quality data, such as bots and fraudulent participants. “Somewhat effective” techniques were those considered capable of identifying a proportion of poor-quality data but that had drawbacks preventing them from being used alone. “No longer effective” techniques were those deemed by the authors of the reviewed articles as being no longer sufficient in addressing threats to data integrity.

When synthesizing the data, we computed the most common types of threats to data integrity, the most common recruitment methods, and the estimated prevalence of threats to data integrity. We also narratively reviewed how authors discussed the adverse effects of threats to data integrity. Next, to address our study objectives, we described all the proposed techniques to address threats to data integrity that we uncovered in this review. Finally, we narratively reviewed how authors discussed the ethical considerations raised by the techniques.

## Results

**Figure 1.**
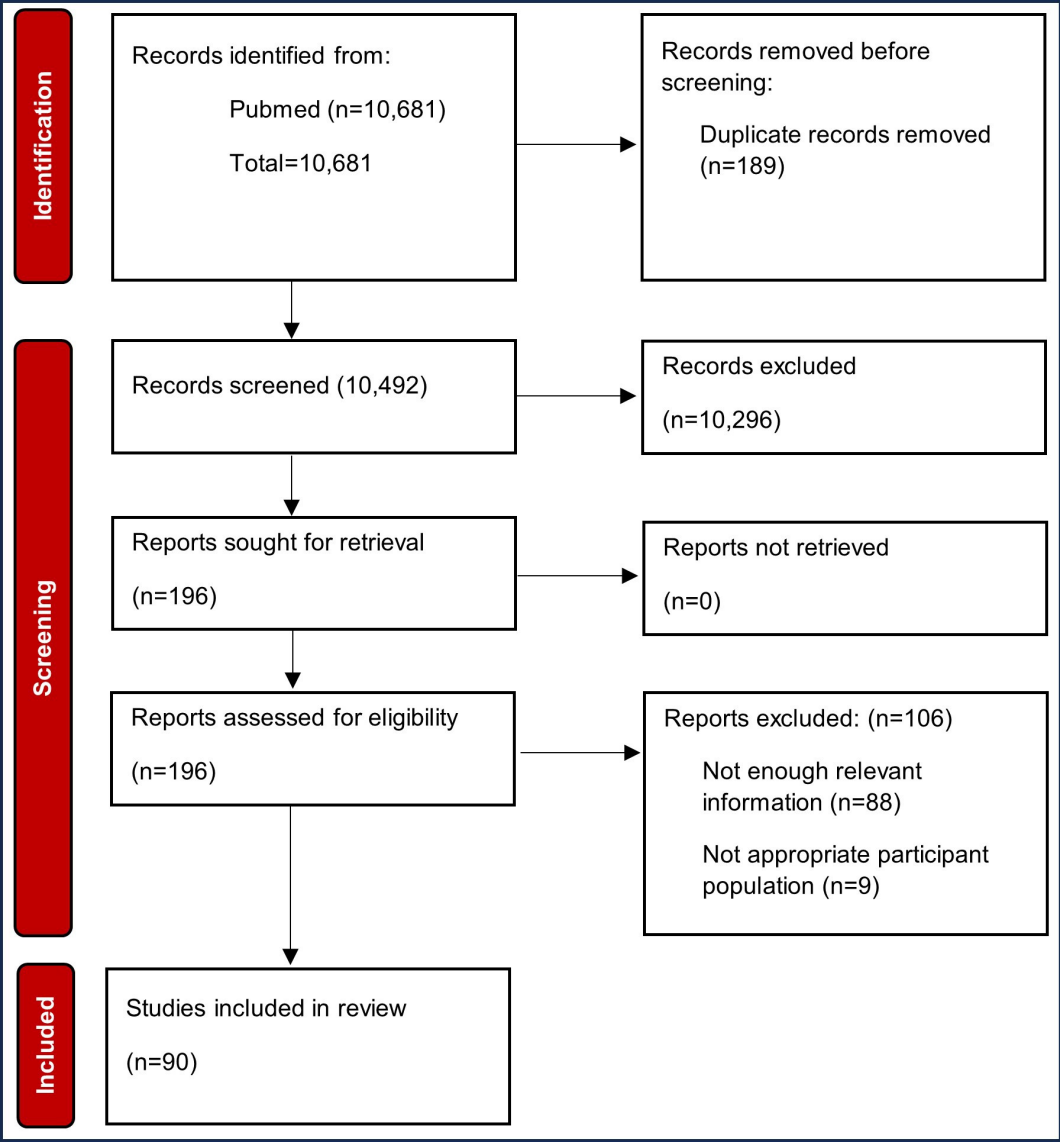
Flow chart of the review process for article selection.

A total of 90 studies were included in the review (Figure 1). The most common type of threat to data integrity documented by researchers was bots (n=59) followed by fraudulent participants (n=51), repeat participants (n=42), and satisficing participants (n=17). The most common recruitment method was social media advertising, followed by using online survey service platforms, such as MTurk (Amazon Web Services, Inc; Table S1 in Multimedia Appendix 1 [2-4,11,13,15,20-103]). Many studies used more than one recruitment method.

The estimated prevalence of threats to data integrity ranged from approximately 1% to 99%. Implications for data validity and reliability were commonly discussed. Other adverse effects included the heavy, and often wasteful, use of resources needed to address fraud. For example, some researchers with a high prevalence of participant fraud described having to end their study and start over, wasting valuable time and resources. Some articles even discussed how dealing with high proportions of imposter participants can be difficult to handle emotionally as researchers. As one research team expressed after finding around 90% of their study participants to be fraudulent: “It is disheartening to encounter issues related to fraud during research. Our team experienced significant demoralization related to this occurrence” [20].

All of the articles provided information on methods to improve the integrity of data, either by (1) preventing the collection of poor-quality data in the first place or (2) identifying and removing poor-quality data if collected (or both; Table S2 in Multimedia Appendix 2 [2-4,11,13,15,20-103]). As cataloged in Table 2, we identified 16 techniques, representing a wide variety of methods. Techniques deemed to be “very effective” included authenticating personal information, such as requesting to see participants’ IDs over a video call, which eliminates the potential for bots and helps identify fraudulent participants. As another version of this technique, Hardesty et al [21] mailed the study incentives to participants’ street addresses (rather than sending the incentive electronically) because they observed that fraudulent participants were providing false addresses to meet geographically based eligibility criteria. A related technique deemed “very effective” was including background-related questions that could be easily answered by participants in the target population (or by partners in dyad research) but that are not widely known by other groups. For example, in a community study on narcolepsy, data were excluded from participants who reported unlikely symptomology [22]. A final “very effective” technique concerned data checking: cross-checking for inconsistent answers (eg, between screening and a baseline survey). Of note, we observed that these very effective techniques were used across a variety of study designs, including both quantitative and qualitative studies.

**Table 2.** Techniques to address threats to data integrity in online research and their frequency of being mentioned across the 90 articles examined in this scoping review.

Effectiveness ranking and technique		Description	Example	Freq.^a^
Very effective				
	Authenticate personal information	Checking IDs, emails, addresses, zip codes, and phone numbers for authenticity. This could include using third-party services to verify identities, requiring video calls at enrollment (verification step), or mailing incentives to the provided street address.	“Include a preinterview briefing over videoconferencing or telephone to go through eligibility criteria and the consent process. Researchers could forewarn potential participants about this aspect in the consent form.” [4]	48.9%
	Background-related questions	Including questions about information that would be easily answered by participants in target population but is not widely known by other groups.	“To reduce fraudulent responses, the study investigators added 4 military validation questions to confirm history of military service prior to the study survey. These questions were developed and piloted with service members and veterans of varying components and across branches.” [23]	20.0%
	Cross-check inconsistent answers	Checking for inconsistent or contradictory answers across survey items to detect fraud or inattention.	“By identifying inconsistencies in data collected at screening and survey data, the team could identify potentially fraudulent or ineligible participants.” [24]	51.1%
Somewhat effective				
	Attention checks	Including survey questions that request specific answers or that may only have one reasonable answer. This screens for satisficing and basic logical reasoning.	“The attention checks consisted of the following: (1) embedded on the Grit scale—“Select ‘Somewhat like me’ for this statement,” (2) embedded on the Beck Depression Inventory—“1 – Select this option,” and (3) embedded on the Borderline Personality Inventory—“Select ‘Yes’ for this statement.’” [25]	33.3%
	Camera-on requirement	Requiring participants to turn on their camera, even if just for a moment, as often fraudulent participants will leave theirs off.	“Participants were not using their cameras for the Zoom sessions because they refused to turn on their camera or they stated there were internet issues.” [26]	10.0%
	Check for dataset duplicates	Checking a dataset for duplicate names, emails, etc, across participants for duplicate replies.	“SAS programs were run to check the newly submitted record against all previous baseline questionnaires to check for duplicates of email addresses, mobile numbers, IP addresses, mailing addresses, social media handles, and preferred names.” [24]	46.7%
	Post hoc testing	Conducting statistical analysis of data for unreasonable response patterns and notable outliers that may be indicative of fraud.	“Interactive visualization can improve data quality by facilitating the identification of issues such as missing data, outliers, duplicates, pattern or constraint violations, and data inconsistencies.” [27]	16.7%
	Watchful of a large number of responses	Checking the timestamps on survey submissions. A flurry of responses or sign-ups can often be an indication of bots or fraud.	“Before launching the DIP, various indications of fraudulent activity were noted. These include…a rush of survey time stamps…found in the same 1‐ to 15-min period.” [28]	30.0%
	Screen for low response rates	Excluding data from participants who complete less than a certain percentage of a study, as they may be satisficing.	“Frequently examine the data for any patterns such as large blocks of blank question.” [29]	8.9%
	Changing payment protocols	Intentionally not emphasizing and not automating participant payments. For example, not including the payment amount in recruitment materials.	“To maximize reach and limit fraud, gift cards could be manually distributed via text or email after each survey is verified.” [30]	21.1%
	Not paying fraudulent respondents	Informing potential participants in the consent form that fraudulent participants will not be compensated. This helps researchers not waste money on fraudulent participants.	“On the consent form, participants were also informed that “…we have put in place a number of safeguards to ensure that participants provide valid and accurate data for this study. If we have strong reason to believe your data are invalid, your responses will not be approved or paid and your data will be discarded.” [31]	11.1%
No longer effective				
	IP address or Geolocation	Examining participant IP and geolocation to see if they match location requirements of study and screening for duplicate IP addresses.	“[Researchers used] other survey platform features to track IP addresses, geolocation, latitude and longitude, and participants’ postal codes when they discovered that geographic markers or indicators did not match the participants’ stated location of residence.” [32]	65.6%
	(re) CAPTCHA	Including tests that can help to screen out bots by providing challenges that theoretically only humans can complete.	“Completely Automated Public Turing test to tell Computers and Humans Apart (CAPTCHA) script was created and implemented into the Google Form.” [33]	45.6%
	Timing checks	Checking for unusually fast or slow response times, which can indicate bots or satisficing.	“We noticed a large proportion of responses with improbably fast completion times (as well as those with particularly long completion times, eg, 4220 min).” [34]	45.6%
	Open-ended questions	Including open-ended questions and reviewing the responses. This can help assess attention as well as check for bots who may incoherently respond.	“Another indicator of data quality is suspicious responses to open-ended questions. For example, when given an open response box to report thoughts or ask questions at the end of the survey, responses written in all caps, one-word responses seemingly unrelated to the prompt, restatements of parts of the question, or nonsensical phrases.” [35]	37.8%
	Honeypots	Incorporating questions into only the code of a survey, such that they are not visible to the human eye. These questions would only be answered by bots.	“We added a honeypot question as a second line of defense against bots. Honeypots are survey questions hidden from rendering on the screen using custom JavaScript code.” [36]	14.4%

a Freq.: frequency of articles mentioning this technique.

Several techniques were deemed “somewhat effective,” as they had notable benefits as well as limitations. For example, attention check questions help detect satisficers and some types of bots but are ineffective against fraudulent participants. Many studies noted suspicion when a large number of surveys were completed at once, as that can indicate their study has been “discovered” by bots or fraudulent respondents; thus, being watchful of a large number of responses is a useful technique but is not sufficient for detecting all cases of threats to data integrity. Another technique, changing payment protocols (eg, intentionally not emphasizing participant payments in recruitment materials), was framed as a preventative measure rather than a definitive means of detecting fraud.

Finally, several techniques were deemed “no longer effective” by the authors: IP address and geolocation checks, reCAPTCHA, timing checks (ie, checking for unusually fast or unusually slow responses), open-ended questions, and honeypot questions (ie, questions not visible to the human eye but would be seen and answered by bots). Although many studies still report using these techniques, many authors also discussed how such methods can now be easily bypassed. For example, reCAPTCHA is not a challenge for most advanced bots, and certainly not for fraudulent participants. Likewise, proxy servers can help “fake” a local IP address. The invention of ChatGPT (OpenAI) and other artificial intelligence natural language processing chatbots makes short answer questions a less effective means of screening, as bots are often able to respond coherently to open-ended questions. Some bots can also be trained to complete surveys in a realistic timeframe and are also able to overlook honeypot questions. Therefore, although they are still somewhat useful for removing simpler bots and fraud attempts, these “no longer effective” techniques are unable to catch or detect more sophisticated attacks and should not be overly relied upon.

While discussing techniques to improve data integrity, many authors reflected on ethical considerations. For instance, as most “very effective” techniques require asking both personal and personally identifiable questions, additional procedures to protect participant privacy and rights may be necessary [37]. Another key ethical concern expressed by researchers was mistakenly excluding genuine participants. For example, fraud detection methods have the potential to introduce selection bias, such as when blocking responses from the same IP address deters residents of high-density housing developments [24]. Deterring genuine participants is also a major concern; for example, many techniques, such as requiring a video call at screening, can place additional burdens on participants and feel invasive [4]. Similarly, verification techniques that convey doubt to participants about their genuineness can compromise trust [38]. As a counter to these concerns, other authors discussed how techniques such as videoconferencing, when conducted with sensitivity, can foster and strengthen rapport and help researchers better understand their participant population [39]. Ultimately, thoughtful study design was encouraged, such as urging researchers to reflect on how to best balance their needs of (1) research integrity and quality; (2) feasibility and efficiency; and (3) safeguarding participants’ rights, safety, and privacy [38]. Often, somewhat minor changes can help work toward this balance. For example, Singh and Sagar [37] encourage methods such as deidentifying data, using encryption processes or password-protected data storage, and using HIPAA (Health Insurance Portability and Accountability Act)-compliant online survey platforms. And Roehl and Harland [5] emphasized the importance of transparency during the consent process, so that participants are aware of what identifiable information will be requested from them and why.

## Discussion

### Principal Findings

Online research is expanding and holds great promise for innovative and impactful research. But as techniques to protect data integrity advance, so too do the methods of mendacious individuals providing false or unreliable responses for monetary gain. In this scoping review, we identified 90 articles published since 2020 that described methods for addressing online threats to data integrity [2-4,11,13,15,20-103]. We found that some of the most common techniques discussed were IP Address or Geolocation checks and reCAPTHCA. This is concerning, given that several articles detailed the reasons these techniques are no longer effective against sophisticated bots or fraudulent participants. Overall, these findings reveal a crucial area for improvement in handling threats to online data integrity. Yet our review also discovered new and innovative techniques for addressing threats to online data integrity. Specifically, we found that authenticating personal information, posing background-related questions, and cross-checking inconsistent answers were deemed very effective techniques for addressing contemporary threats.

### Recommendations

While there is no one foolproof way for researchers to prevent participant fraud, it is clear from this review that the field has moved beyond reCAPTCHA as a sufficient technique for ensuring data integrity. Bots are advancing and fraudulent participants are becoming more sophisticated, making reCAPTCHA ill-equipped to handle the scope of the current problem. We recommend that researchers engaging in online data collection develop a robust strategy for ensuring data integrity early in the design of their research protocol. For such designs, we recommend that researchers use multiple techniques (rather than relying on the soundness of just one technique), use the techniques described in the articles reviewed here, and draw the most from techniques deemed to be “very effective.” Although the constraints of timelines, person power, and budgets can render some techniques unfeasible, many “very effective” techniques are efficient and low cost; for example, researchers can think creatively to develop background-related questions that, even with internet searching, would only be readily known and (or) accurately answered by their population. When relying on survey service platforms for access to online samples, researchers should be critical of the techniques used by the platforms to guarantee the quality of their panels. We also suggest that techniques for ensuring data integrity should be critically considered by journal editors, journal reviewers, and grant reviewers when evaluating the rigor of study methods.

The ethical concerns discussed by the articles in this review highlight the responsibilities of researchers to continue focusing on participant rights and privacy. Techniques for ensuring data integrity (eg, personal questions to authenticate identity) should be balanced against these responsibilities. More broadly, the comfort of participants and their rapport with the study team should be considered. The relative weight of these considerations will vary depending on many factors. For instance, more complex study designs (eg, longitudinal studies that rely on participant trust and investment for good retention) may require techniques that integrate authentication checks with rapport-building. The vulnerability of the sample and the sensitivity of the research topic must also be considered. Techniques for improving data integrity that increase participant burden, barriers, or privacy risk must be matched with greater participant accommodations—this could include greater compensation, clear explanations to participants during consent and enrollment about why certain questions are being asked, and enhanced data protection measures. Researchers attempting to exclude fraudulent participants should always be aware of their own biases and ensure that they are not excluding participants simply because they do not align with expected results.

### Limitations and Future Directions

Our scoping review has some limitations. First, we only used one database for review (PubMed). This was done due to the large volume of articles on the subject and our focus on health research; however, it may have overlooked insights from other fields. Second, our search terms led to the studies reviewed primarily focusing on bots, fraudulent participants, and repeat participants, likely leading to an overestimation of these behaviors and an underestimation of the prevalence of participant satisficing and the tactics used to mitigate those behaviors. It is important to acknowledge that moving forward, some tactics that are currently in our “very effective” category may become less effective with the evolution of artificial intelligence, or as fraudulent participants become more familiar with current strategies. Going forward, more empirical studies should be conducted on research methods for addressing threats to data integrity, to quantitatively compare the effectiveness of various techniques used to address threats to data integrity in online research. Future work should also consider the participant perspective on these various techniques in order to improve their effectiveness and minimize negative consequences.

### Conclusions

Threats to data integrity appear to be on the rise, particularly with online research, and numerous solutions and prevention strategies have been recommended. In order to aid researchers in developing robust online methodologies, this scoping review discusses the most common types of threats to data integrity, synthesizes the most common prevention methods, and discusses the ethical considerations raised about the techniques. Doubtless, new threats to data integrity will continue to emerge, and researchers should continue developing the most effective methods in response.

## Supplementary material

10.2196/70926 Multimedia Appendix 1 Study details described in the 90 manuscripts examined in this scoping review.

10.2196/70926 Multimedia Appendix 2 Techniques for addressing threats to data integrity in online research as mentioned in the 90 manuscripts examined in this scoping review.

10.2196/70926 Checklist 1 PRISMA (Preferred Reporting Items for Systematic Reviews and Meta-Analyses) checklist.

## Abbreviations

HIPAA: Health Insurance Portability and Accountability Act
PRISMA: Preferred Reporting Items for Systematic Reviews and Meta-Analyses
