# Supplementary material for: Imposters, Bots, and Other Threats to Data Integrity in Online Research: Scoping Review of the Literature and Recommendations for Best Practices
Source: Online J Public Health Inform. 2025 Aug 29;17:e70926. doi: 10.2196/70926 (PMC12396152; doi:10.2196/70926)
Supplement: Multimedia Appendix 1 [file ojphi-v17-e70926-s001.docx]

**Table S1.**

| Article Name | Authors and Year of Publication | Article type | Type of Data Collection | Methodology | Country in which the Study was Conducted | Recruitment Method | Estimated Prevalence of Data Integrity Threat | Type(s) of Threat to Data Integrity Mentioned |
| --- | --- | --- | --- | --- | --- | --- | --- | --- |
| Quality control questions on Amazon’s Mechanical Turk (MTurk): A randomized trial of impact on the USAUDIT, PHQ-9, and GAD-7 | (Agley et al 2021) | Original Research | Quantitative | Survey | United States | Survey Service Platform | The approach to identifying users potentially using VPNs or bots to complete surveys generated fewer rejections (n = 55) than the questions to catch inattentive or dishonest respondents (n = 189), while the arm with both control types excluded even more (n = 210) | Bots, Fraudulent Participants, Satisficers |
| Recruiting Nurses Via Social Media for Survey Studies. | (Bethel et al 2021) | Original Research | Quantitative and Qualitative | Survey | United States | Social Media, Listservs, Snowball Sampling | / | Bots |
| Artificial Intelligence in Medicine: A Multinational Multi-Center Survey on the Medical and Dental Students' Perception | (Bisdas et al 2021) | Original Research | Quantitative and Qualitative | Survey | Respondents from 63 countries from all continents were included | Social Media | / | Fraudulent Participants, Bots, Repeat Participants |
| Assessing and Improving Data Integrity in Web-Based Surveys: Comparison of Fraud Detection Systems in a COVID-19 Study. | (Bonett et al 2024) | Original Research | Quantitative | Survey | United States | Social Media, Community Partners | 59.40% of survey responses classified as fraud. | Bots, Fraudulent Participants, Repeat Participants |
| Concerns and recommendations for using Amazon MTurk for eating disorder research. | (Burnette et al 2022) | Original Research | Quantitative and Qualitative | Survey | United States | Survey Service Platform | >90% of data deemed invalid and research project abandoned. | Bots, Fraudulent Participants, Satisficers, Repeat Participants |
| Social Media as a Recruitment Strategy with Transgender-Identified Individuals: Using an Ethical Lens to Direct Methodology. | (Bush & Blackwell 2022) | Original Research | Quantitative | Survey | United States | Social Media | / | Bots |
| Bots and nots: Safeguarding online survey research with underrepresented and diverse populations | (Bybee et al 2022) | Original Research | Quantitative | Survey | United States | Social Media, Snowball Sampling | >90% of interest forms not eligible. Study was halted. | Bots, Fraudulent Participants, Repeat Participants |
| Overcoming Challenges of Online Research: Measures to Ensure Enrollment of Eligible Participants. | (Campbell et al 2022) | Original Research | / | Survey | United States | Social Media | 413/1308= 55.8% | Bots, Fraudulent Participants, Repeat Participants |
| Participant carelessness and fraud: Consequences for clinical research and potential solutions. | (Chandler et al 2020) | Commentary | / | Survey | United States | Survey Service Platform | Approximately 3-7% of responses on MTurk surveys for US participants associated with non-US IP addresses. Fraud rates variable ranging from 3-40% | Satisficers |
| Integrating statistical and visual analytic methods for bot identification of health-related survey data. | (Chen et al 2023) | Original Research | Quantitative and Qualitative | Survey | United States | Social Media, Listservs | ~80% of responses excluded based on fraud prevention criteria | Bots |
| The impact of narcolepsy on social relationships in young adults. | (Davidson et al 2022) | Original Research | Quantitative | Survey | United States | Listservs | / | Fraudulent Participants |
| Management of fraudulent participants in online research: Practical recommendations from a randomized controlled feasibility trial. | (Davies et al 2024) | Original Research | Quantitative and Qualitative | Survey | UK | Social Media | From the first bot attack ~96% of responses found to be fraudulent. 100% of participants during the second attack found to be fraudulent. Participants between and before attacks all legitimate. | Bots, Fraudulent Participants, Repeat Participants |
| Data quality in online human-subjects research: Comparisons between MTurk, Prolific, CloudResearch, Qualtrics, and SONA. | (Douglas et al 2023) | Original Research | Quantitative and Qualitative | Survey | United States | Survey Service Platform, Student Subject Pool | Percentage of responses that were not "high quality" were: 73.6% for MTurk, 38.0% for CloudResearch, 32.0% for Prolific, 46.8% for Qualtrics, and 47.2% for the Student Subject Pool | Fraudulent Participants, Satisficers |
| Beyond the challenge to research integrity: imposter participation in incentivised qualitative research and its impact on community engagement. | (Drysdale et al 2023) | Commentary | / | Survey, Interview | Australia | / | An interview recruiting 30 participant received 469 emails from suspected imposter participants. Another got 80 responses approximately 30 of which were from suspected imposter participants. | Fraudulent Participants, bots |
| Contrasting a Mobile App With a Conversational Chatbot for Reducing Alcohol Consumption: Randomized Controlled Pilot Trial | (Dulin et al 2022) | Original Research | Quantitative | Survey | United States | Social Media | / | Fraudulent Participants |
| Modeling the Probability of Fraud in Social Media in a National Cannabis Survey | (Dutra et al 2021) | Original Research | Quantitative | Survey | United States | Social Media | 6,370 with a 50% or higher probability of fraud | Bots, Fraudulent Participants, Repeat Participants |
| Latinas in medicine: evaluating and understanding the experience of Latinas in medical education: a cross sectional survey. | (Geiger et al 2024) | Original Research | Quantitative | Survey | United States | Social Media, Listservs | / | Fraudulent Participants |
| Liar! Liar! Identifying eligibility fraud by applicants in digital health research. | (Glazer et al 2021) | Original Research | Quantitative and Qualitative | Survey, Interview | United States | Social Media, Flyers | Of 1766 total interest forms received, 125 (7.08%) were fraudulent | Fraudulent Participants, Repeat Participants |
| Out damn bot, out: Recruiting real people into substance use studies on the internet. | (Godinho et al 2020) | Commentary | / | / | Canada, Australia | / | 123 (16.0%) did not provide real addresses, 15 (1.7%) provided incomplete addresses and could not be contacted, and 15 (1.7%) attempted to enroll into the study more than once. | Repeat Participants, Bots |
| Did a bot eat your homework? An assessment of the potential impact of bad actors in online administration of preference surveys. | (Gonzalez et al 2023) | Original Research | Quantitative | Survey | United States | Survey Service Platform | / | Bots, Fraudulent Participants, Repeat Participants |
| Fraudulent Online Survey Respondents May Disproportionately Threaten Validity of Research in Small Target Populations. | (Gordon et al 2024) | Commentary | Quantitative | Survey | United States | Social Media | 1487/1843 (~80%) | Fraudulent Participants, Bots, Repeat Participants |
| Prospective interactive influence of financial strain and emotional nonacceptance on problematic alcohol use during the COVID-19 pandemic. | (Gratz et al 2021) | Original Research | Quantitative | Survey | United States | Survey Service Platform | At baseline, participants who failed an attention check were removed from the study (n = 53 of 553) | Satisficers |
| Thwarted belongingness and perceived burdensomeness explain the associations of COVID-19 social and economic consequences to suicide risk. | (Gratz et al 2020) | Original Research | Quantitative and Qualitative | Survey | United States | Survey Service Platform | 53/553= 9.6% | Bots |
| Ensuring survey research data integrity in the era of internet bots. | (Griffin et al 2022) | Original Research | Quantitative and Qualitative | Survey | United States | Social Media, Listservs | 86% of respondents identified as bots | Bots, Fraudulent Participants |
| Adapting a selective parent-focused child sexual abuse prevention curriculum for a universal audience: A pilot study. | (Guastaferro et al 2024) | Original Research | Quantitative and Qualitative | Survey, Interview, Workshop | United States | Social Media | 174/268 (~65) | Bots |
| Methods for Authenticating Participants in Fully Web-Based Mobile App Trials from the iReach Project: Cross-sectional Study. | (Guest et al 2021) | Original Research | Quantitative | Survey | United States | Social Media, Flyers | ~50% of participants excluded due to suspected fraud | Fraudulent Participants, Bots, Repeat Participants |
| AIM against survey fraud. | (Habib and Jha 2021) | Original Research | / | Survey | United States | Direct Contact | Listserv | Fraudulent Participants, Repeat Participants |
| The Vaping and Patterns of e-Cigarette Use Research Study: Protocol for a Web-Based Cohort Study. | (Hardesty et al 2023) | Original Research | Quantitative | Survey | United States | Social Media | 77.6% invalid survey completions in failed attempt before wave 1 | Bots, Fraudulent Participants, Repeat Participants |
| Do you know the Wooly Bully? Testing era-based knowledge to verify participant age online. | (Hartman et al 2023) | Original Research | Quantitative | Survey | United States | Survey Service Platform | 72% of participants known to be younger than the wanted demographic claimed to be 50 or older. The tool caught of 80% of these participants. | Fraudulent Participants |
| Evaluating CloudResearch's Approved Group as a solution for problematic data quality on MTurk. | (Hauser et al 2023) | Original Research | Quantitative and Qualitative | Survey | United States | Survey Service Platform | Estimated more than 1/3 | Bots, Fraudulent Participants, Satisficers |
| Preventing and Protecting Against Internet Research Fraud in Anonymous Web-Based Research: Protocol for the Development and Implementation of an Anonymous Web-Based Data Integrity Plan. | (Hohn et al 2022) | Original Research | Quantitative and Qualitative | Survey, Interview | United States | / | 3/12 enrolled participants found to be fraudulent | Satisficers, Repeat Participants |
| Study Protocol and Preliminary Results of the Impact of Occupational Health Workers' Activities on Their Health: Nationwide Prospective Internet-Based Survey. | (Ikegami et al 2022) | Original Research | Quantitative | Survey | Japan | Listservs | ~12% | Satisficers |
| Model-agnostic unsupervised detection of bots in a Likert-type questionnaire. | (c et al 2023) | Original Research | Quantitative | / | Canada | Simulation Data | / | Bots |
| Supervised Classes, Unsupervised Mixing Proportions: Detection of Bots in a Likert-Type Questionnaire. | (Ilagan and Falk 2023) | Original Research | Quantitative | Survey | Canada | Simulation Data | / | Bots |
| A Web-Based, Respondent-Driven Sampling Survey Among Men Who Have Sex With Men (Kai Noi): Description of Methods and Characteristics. | (Karuchit et al 2024) | Original Research | Quantitative and Qualitative | Survey, Interview, Biometrics | Thailand | Social Media | 15.00% | Repeat Participants |
| Too Anecdotal to Be True? Mechanical Turk Is Not All Bots and Bad Data: Response to Webb and Tangney (2022) | (Keith and McKay 2024) | Commentary | / | / | United States | Survey Service Platform | / | Bots, Satisficers |
| Challenges and Lessons Learned From a Mobile Health, Web-Based Human Papillomavirus Intervention for Female Korean American College Students: Feasibility Experimental Study. | (Kim et al 2020) | Original Research | Quantitative | Survey | United States | Social Media | 43/225= (~19%) | Fraudulent Participants, Repeat Participants |
| Measuring psychiatric symptoms online: A systematic review of the use of inventories on Amazon Mechanical Turk (mTurk). | (Kolc et al 2023) | Review | / | Literature Search | Australia | Survey Service Platform | / | Bots, Satisficers |
| Evaluating the Problem of Fraudulent Participants in Health Care Research: Multimethod Pilot Study. | (Kumarasamy et al 2024) | Original Research | Quantitative and Qualitative | Survey, Interview | Canada | Listservs | total of 84% (31/37) participants indicated that fraudulent participation occurred in studies that mentioned incentives in their recruitment | Bots, Fraudulent Participants, Repeat Participants |
| Methodological Challenge: Addressing Bots in Online Research. | (Lawrence et al 2023) | Methodology Paper | Quantitative and Qualitative | Survey | United States | Social Media, Listservs, Flyers | 11/109 legitimate= (~90%) fraudulent, second study 100% of responses found to be fraudulent, | Bots |
| Online randomised trials with children: A scoping review. | (Lepage et al 2023) | Review | Qualitative | Literature Search | UK | / | / | Fraudulent Participants, Bots, Repeat Participants |
| Survey Fraud and the Integrity of Web-Based Survey Research. | (Levi et al 2022) | Commentary | / | Survey | United States | Social Media, Flyers | excluded 228 of the 482 surveys (47.3%)) | Bots, Fraudulent Participants |
| Bot or Not? Detecting and Managing Participant Deception When Conducting Digital Research Remotely: Case Study of a Randomized Controlled Trial. | (Loenberg et al 2023) | Original Research | Quantitative | Survey | UK | Social Media, Flyers, Radio | 75.6% of participants identified as bots during data screening, 5% of participants enrolled in the study identified as false participants | Bots, Fraudulent Participants |
| Sociodemographics and Transdiagnostic Mental Health Symptoms in SOCIAL (Studies of Online Cohorts for Internalizing Symptoms and Language) I and II: Cross-sectional Survey and Botometer Analysis. | (Lorenzo-Luaces et al 2022) | Original Research | Quantitative | Survey | United States | Survey Service Platform | in SOCIAL 1, deemed bot-like (190/1123, 16.91%). In SOCIAL 2, smaller fraction deemed bot-like (229/1228, 18.65%) | Bots |
| Recommendations From a Descriptive Evaluation to Improve Screening Procedures for Web-Based Studies With Couples: Cross-Sectional Study. | (Mitchell et al 2020) | Original Research | Quantitative | Survey | United States | Social Media | 450 paired individuals (225 dyads) were identified as fraudulent. Of these 3815 individuals, 2279 were excluded (59.7%) | Fraudulent Participants, Repeat Participants |
| Identifying and Mitigating Fraud When Using Social Media for Research Recruitment. | (Mizerek 2023) | Original Research | Qualitative | Interview | United States | Social Media | ~90% rate of fraudulent participants | Fraudulent Participants |
| Internet-Based Mental Health Survey Research: Navigating Internet Bots on Reddit. | (Mournet and Kleiman 2023) | Original Research | Quantitative | Survey | United States | Social Media | 33.4% of participant found to have come from just 8 locations indicating bot farms | Bots |
| Reaching the "Hard-to-Reach" Sexual and Gender Diverse Communities for Population-Based Research in Cancer Prevention and Control: Methods for Online Survey Data Collection and Management. | (Myers et al 2022) | Original Research | Quantitative and Qualitative | Survey | United States | Direct Mail, Social Media, Flyers, Listservs, Community Outreach | ~27% duplicate responses likely completed by bots. 16% of responses fraudulent | Bots, Repeat Participants |
| Fraudulent participants in qualitative child health research: identifying and reducing bot activity. | (O'Donnell et al 2023) | Commentary | / | Survey, Interview | UK | Social Media | 385/483 fraudulent participants using enhanced screening (80%) | Bots, Fraudulent Participants |
| "Taking out the trash": Strategies for preventing and managing fraudulent data in web-surveys. | (Panesar et al 2023) | Commentary | / | Survey | Canada | / | / | Bots, Fraudulent Participants, Repeat Participants |
| Lessons Learned Recruiting and Retaining Pregnant and Postpartum Individuals in Digital Trials: Viewpoint. | (Parks et al 2022) | Methodology Paper | Quantitative and Qualitative | Survey, Interview | Australia, United States | Social Media | / | Bots, Fraudulent Participants, Repeat Participants |
| Social Media Recruitment Strategies to Recruit Pregnant Women Into a Longitudinal Observational Cohort Study: Usability Study. | (Pekarsky et al 2022) | Original Research | Quantitative | Survey | Canada | Social Media, Flyers | 78% of participants deemed fraudulent | Fraudulent Participants |
| Letter to the Editor: A possible threat to data integrity for online qualitative autism research. | (Pellicano et al 2024) | Commentary | Qualitative | Interview | Australia, United States, UK | Community Partners | / | Fraudulent Participants |
| Mischief-making bots attacked my scientific survey. | (Perkel 2020) | Commentary | Quantitative | Survey | United States | Social Media | / | Bots |
| Threats of Bots and Other Bad Actors to Data Quality Following Research Participant Recruitment Through Social Media: Cross-Sectional Questionnaire. | (Pozzar et al 2020) | Original Research | Quantitative | Survey | United States | Social Media | 94.5% of responses classified as fraudulent | Bots, Satisficers, Repeat Participants |
| Strategies for the Identification and Prevention of Survey Fraud: Data Analysis of a Web-Based Survey. | (Pratt-Chapman et al 2021) | Original Research | Quantitative | Survey | United States | Social Media, Listservs | 71.2% (1408/1977) | Bots, Repeat Participants |
| The Cyborg Method: A Method to Identify Fraudulent Responses from Crowdsourced Data. | (Price et al 2024) | Original Research | Quantitative | Survey | United States | Survey Service Platform | sample 1: of the 4427 responses, 1815 (61.7%) had invalid IP's and another 250 were deemed invalid due to their open-ended responses. | Bots |
| Combating Fraudulent Participation in Urban American Indian and Alaska Native Virtual Health Research: Protocol for Increasing Data Integrity in Online Research (PRIOR). | (Reed et al 2024) | Original Research | Quantitative | Survey | United States | Social Media, Listservs | ~99% of participant fraudulent | Bots, Fraudulent Participants, Repeat Participants |
| Imposter participants' in online qualitative research, a new and increasing threat to data integrity? | (Ridge et al 2023) | Commentary | Qualitative | Interview | UK | Social Media | / | Fraudulent Participants |
| Creating a Bot-tleneck for malicious AI: Psychological methods for bot detection. | (Rodriguez and Oppenheimer 2024) | Original Research | Quantitative | Survey | United States | Survey Service Platform | 18.9% of the sample identified as bots | Bots |
| Digital Global Recruitment for Women's Health Research: Cross-sectional Study. | (Rodriguez et al 2022) | Original Research | Quantitative | Survey | United States | Social Media | 46.2% of consents filled out by bot | Bots |
| Automated Bot Detection Using Bayesian Latent Class Models in Online Surveys. | (Roman et al 2022) | Original Research | Quantitative | Survey | Switzerland, Germany, United States | Survey Service Platform | / | Bots |
| Utilization of online systems to promote youth participation in research: A methodological study. | (Salem et al 2023) | Methodology Paper | Quantitative | Survey | United States | Flyers |  | Bots, Fraudulent Participants, Repeat Participants |
| Are Your Participants Real? Dealing with Fraud in Recruiting Older Adults Online. | (Salinas 2023) | Methodology Paper | Quantitative | Survey | United States | Survey Service Platform, Social Media | 2/46= 4.3% MTurk, 176/186= 95% on facebook link 1, 44/80=55% from facebook link 2, 179/183= 98% from facebook link 3, 0/23 from email | Bots, Fraudulent Participants, Repeat Participants |
| When snowball sampling leads to an avalanche of fraudulent participants in qualitative research | (Sefcik et al 2023) | Original Research | Qualitative | Interview | United States | Listservs, Flyers, Snowball Sampling | Researchers ended up excluding all participants in first round due to high fraud. | Fraudulent Participants |
| Navigating the challenges of imposter participants in online qualitative research: lessons learned from a paediatric health services study. | (Sharma et al 2024) | Original Research | Qualitative | Interview | Australia | Social Media | Researchers ended up excluding all participants in first round due to high fraud. | Fraudulent Participants |
| Detecting possible persons of interest in a physical activity program using step entries: Including a web-based application for outlier detection and decision-making. | (Silva et al 2020) | Original Research | Quantitative | Survey, Biometrics | Australia | Secondary Data | persons of interest: 687 (1.45%) | Fraudulent Participants |
| A quasi-experimental study examining the efficacy of multimodal bot screening tools and recommendations to preserve data integrity in online psychological research. | (Simone et al 2023) | Original Research | Quantitative | Survey | United States | Social Media | From 1.1% to 81.3%, depending on the criteria | Bots, Fraudulent Participants |
| A critical look at online survey or questionnaire-based research studies during COVID-19. | (Singh and Sagar 2021) | Commentary | / | Survey | India | Social Media |  | Satisficers, Repeat Participants |
| Smartphone-based Respondent Driven Sampling (RDS): A methodological advance in surveying small or 'hard-to-reach' populations. | (Sosenko and Bramley 2022) | Original Research | Quantitative | Survey | UK | Direct Contact, Snowball Sampling | 102/422= (~25%) fraudulent | Fraudulent Participants, Satisficers, Repeat Participants |
| Correlates of Successful Enrollment of Same-Sex Male Couples Into a Web-Based HIV Prevention Research Study: Cross-Sectional Study. | (Stephenson et al 2020) | Original Research | Quantitative | Survey | United States | Social Media | 12.86% fraudulent | Fraudulent Participants, Repeat Participants |
| Using Military Screening Questions to Anonymously Recruit Post-9/11 Era Service Members and Veterans Using Online Survey Methods. | (Tannahill and Blais 2024) | Original Research | Quantitative | Survey | United States | Survey Service Platform | 92% deemed likely fraudulent | Bots, Fraudulent Participants |
| Geolocation to Identify Online Study-Eligible Gay, Bisexual, and Men who have Sex with Men in Philadelphia, Pennsylvania. | (Tran et al 2023) | Original Research | Quantitative | Survey | United States | Social Media | According to a 4-step approach:47%; according to their geolocation algorithm: 42% | Bots, Fraudulent Participants, Repeat Participants |
| Using Google Ads to recruit and retain a cohort considering abortion in the United States. | (Upadhyay et al 2020) | Methodology Paper | Qualitative | Survey | United States | Social Media | 21/1,485= (~1%) | Repeat Participants |
| Participant Preferences for the Development of a Digitally Delivered Gardening Intervention to Improve Diet, Physical Activity, and Cardiovascular Health: Cross-sectional Study. | (Veldheer et al 2023) | Original Research | Quantitative and Qualitative | Survey | United States | Survey Service Platform | 1st screening= 91/808= 11.3%. 2nd screening= 250/717= 34.9% | Bots, Satisficers |
| Satisficing and bots. | (Venugopal et al 2022) | Commentary | / | Survey | India, Cambodia, Germany, Saudi Arabia | Social Media, Listservs | ~60% | Bots, Satisficers, Repeat Participants |
| Experience and lessons learned from multi-modal internet-based recruitment of U.S. Vietnamese into research. | (Vu et al 2021) | Original Research | Quantitative and Qualitative | Survey, Interview | United States | Social Media, Listservs, Snowball Sampling | / | Bots, Fraudulent Participants |
| Improving Data Integrity and Quality From Online Health Surveys of Women With Infant Children. | (Walker et al 2023) | Original Research | Quantitative | Survey | United States | Survey Service Platform, Social Media, Listservs, Flyers | ~55% | Bots, Fraudulent Participants, Satisficers, Repeat Participants |
| Identifying and preventing fraudulent responses in online public health surveys: Lessons learned during the COVID-19 pandemic. | (Wang et al 2023) | Original Research | Quantitative | Survey | United States | Social Media, Flyers, Listservs | / | Bots, Fraudulent Participants, Repeat Participants |
| Conducting conservation social science surveys online. | (Wardropper et al 2021) | Methodology Paper | / | Survey | United States | Survey Service Platform, Listservs |  | Bots, Satisficers |
| Too Good to Be True: Bots and Bad Data From Mechanical Turk. | (Webb and Tangney 2022) | Commentary | Quantitative and Qualitative | Survey | United States | Survey Service Platform | ~95% | Bots |
| The detection and management of attempted fraud during an online randomised trial. | (Willis et al 2023) | Original Research | Quantitative | Survey | UK | Listservs | 268/603 = 44% | Repeat Participants |
| A videoconferencing verification method for enrollment of breastfeeding dyads to an online prospective mixed methods study during the COVID-19 pandemic. | (Wood and Bindler 2023) | Original Research | Quantitative | Survey | United States | Social Media, Word of Mouth, Flyers | 43/109= (~40%) | Bots, Repeat Participants |
| Identifying and preventing fraudulent participation in qualitative research. | (Woolfall 2023) | Methodology Paper | / | Interviews | UK | Social Media | / | Fraudulent Participants, Repeat Participants |
| Participant Fraud in Virtual Qualitative Substance Use Research: Recommendations and Considerations for Detection and Prevention Based on a Case Study. | (Wright et al 2024) | Commentary | Qualitative | Interview | Canada | Social Media, Flyers | 27/50= (~54%) | Fraudulent Participants, Bots |
| The Public's Self-Avoidance and Other-Reliance in the Reporting of Medical Insurance Fraud: A Cross-Sectional Survey in China. | (Xu et al 2023) | Original Research | Quantitative | Survey | China | unclear | 43/900 = 4.8% | / |
| What influences the public's willingness to report health insurance fraud in familiar or unfamiliar healthcare settings? a cross-sectional study of the young and middle-aged people in China. | (Xu et al 2024) | Original Research | Quantitative | Survey | China | unclear | 63/900 = 7% | / |
| Novel Recruitment Methods for Research Among Young Adults in Rural Areas Who Use Opioids: Cookouts, Coupons, and Community-Based Staff. | (Young 2020) | Original Research | Quantitative | Survey | United States | Flyers, Snowball Sampling | 67/234=(~30%) | Bots, Repeat Participants |
| Who is More Likely to Report Medical Insurance Fraud in the Two Scenarios of Whether It Results in a Direct Loss of Individual Benefit? A Cross-Sectional Survey in China. | (Zhang et al 2022) | Original Research | Quantitative | Survey | China | unclear | 57/628= (~9%) | Repeat Participants |
| Recruiting foreign-born individuals who have sought an abortion in the United States: Lessons from a feasibility study. | (Zuniga et al 2023) | Original Research | Quantitative | Survey, Interview | United States | Survey Service Platform, Social Media | 103/900= (~10%) | Repeat Participants |
